# Supplementary material for: Optogenetic spatial patterning of cooperation in yeast populations
Source: Nat Commun. 2024 Jan 2;15:75. doi: 10.1038/s41467-023-44379-5 (PMC10761962; doi:10.1038/s41467-023-44379-5)
Supplement: Supplementary file 1 — Supplementary Information [file 41467_2023_44379_MOESM1_ESM.pdf]

Optogenetic spatial patterning of cooperation in yeast populations

Le Bec *et al.*

Supplementary Method 1. Building the OptoCube

Building an OptoCube is relatively straightforward. We used a static incubator with relatively large dimensions, a Digital micromirror device, a microcontroller, and a flatbed scanner. We propose here a set of devices that worked for us, but other commercial or DIY alternatives are of course possible. Details and scripts can be found here: https://github.com/Lab513/DIY_OptoCube. Briefly, the DMD was mounted on an incubator rack (OpenBeam construction kits). The scanner was placed below, facing up. The distance between the scanner glass and the DMD lens was 39 cm.

- Incubator: Memert (any sufficiently large incubator will do)
- DMD: DLP® LightCrafter™ 4500 TI
- Scanner: Canon LIDE400. Note that we opened the glass cover and applied black tape on some parts of the bottom of the scanner that are reflecting surfaces (this prevents undesired optogenetic activation due to reflected light artefacts).
- Microcontroller: Arduino UNO.

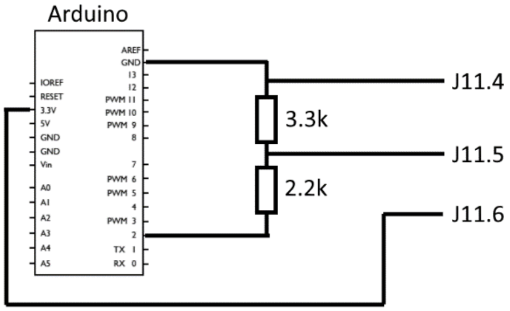

Controlling the DMD with the Arduino board

The DMD was powered with a 12 V 4.16 A power supply and connected to the Arduino by pins J11.4, J11.5, and J11.6 using a molex 51021-0600 connector. The scanner was connected to the computer with a USB cable. The Arduino UNO was also connected to the computer with a USB cable and used to control the DMD via pin number 2 to apply 3.3 V at the J11.5 pin through a voltage divider (2.2 kOhm and 3.3 kOhm). The Arduino 3.3 V pin was connected to the J11.4 pin and the ground pin, to the J11.6. We used Jupyter notebook to drive the OptoCube. The following packages were installed:

- serial (https://pypi.org/project/pyserial/)
- time (https://docs.python.org/fr/3/library/time.html)
- os (https://docs.python.org/fr/3/library/os.html).

The DLP® LightCrafter™ 4500 TI is a digital micromirror device (DMD) composed of 912 × 1140 micromirrors that can switch ON or OFF to reflect the light emitted from integrated LEDs (red, green, and blue). The projected intensity of each micromirror is controlled by pulse width modulation. One important technical limitation is that even when the mirrors are completely OFF, a significant amount of light leaks out of the DMD onto the projected surface. The DMD offers two main modes of projection: video mode or sequence mode. Video mode is performed through classical HDMI communication with a computer, allowing for straightforward and dynamic patterning. However, the range of light intensity in video mode is quite low, with high leakage. Thus, we used the sequence mode, which provides a better intensity range (from 0.0014 mW.cm^{-2} to 1.13 mW/.cm^{-2} for the blue LED). With this setting, we did not observe significant activation of the EL222 system in the OFF mode.

The main drawback of this mode is the lack of flexibility to change the mask being projected by the DMD. The mask must be loaded in the DMD before the experiment starts and the maximum number of 8-bit masks that can be stored is six. We used the DLPLCR software ("DLPLCR4500EVM-GUI", which can be downloaded here https://www.ti.com/tool/DLPLCR4500EVM) to create and load the masks and to design the pattern sequence. More information on the procedure can be found in the DMD user guide. Briefly, the workflow is as follows:

1. Create an 8-bit image in BMP format of dimensions 912 x 1140 (width x height). The pattern must be drawn with 200% deformation in the vertical axis (due to the diamond shape of the mirrors; see the DLPLCR documentation for details)
2. This image is transformed by the DLPLCR software to a 24-bit BMP image.
3. Load the 24-bit image to the firmware using the DLPLCR software.
4. Connect the computer to the DMD using a USB cable and power the DMD.
5. Load the firmware in the DMD.
6. Edit and save the pattern time sequence in the DMD. You can then unplug the computer from the DMD.

Setting up the VueScan© software to drive the scanner
We used the following parameters:

- Media: "Black&White"
- Media size: A4
- Output file: 16-bit greyscale .tiff (no reduction nor compression)
- Make Grey from: Auto
- Scan resolution: 600 dpi
- Number of passes: 1
- Color balance: "None"

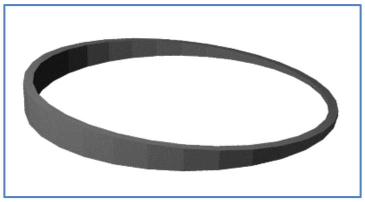

Requirements

The Petri dish lids can generate imaging artefacts by reflecting light. To prevent this, we used a specially designed 3D printed part ("PlateTilterAngle5") to tilt the lids of the Petri dishes at 5°. Note that to maximize the image quality, you should ensure that the agar plates are:

1. Highly transparent to avoid light scattering (this is why we relied on Phytagel).
2. The gel layer is thin to allow the yeast layer to be in the focal plan of the scanner.
3. The lid should be coated with a surfactant solution to reduce droplet formation due to condensation. We used Triton 100X 0.05% (*v/v*) in 20% ethanol.

Supplementary Method 2. Model of the cooperator/cheater system

To model the growth of yeast in sucrose, we use Michaelis–Menten kinetics for the enzymatic reactions (invertase hydrolysis Eq (**3**)) and high and low affinity glucose transporter Eq (**4**) and the Monod equation for the yeast growth rate Eq (**6**). Cells can be either cooperator or cheater, depending on the presence of light. They have distinct invertase production rate α: 1.8E-24 mol.s-1.cell-1 for cooperators (at maximum light induction) and 1.5E-25 mol.s-1.cell-1 for cheaters. For intermediate light stimulation α is linearly computed between these two values. Note that dE/dt (rate of production of the invertase) depends on the hexose concentration following a Michaelis-Menten relationship. Thus, as expected from the literature, significant amount of invertase is produced at low glucose (50% of the maximal production rate at 0.0002% glucose). Also, this relationship prevents the invertase concentration to increase indefinitely after the nutrients are fully depleted.

$$\frac{dS}{dt}(x) = D_S \frac{d^2S}{dx^2} - I(x) \quad \textbf{(1)}$$

$$\frac{dM}{dt}(x) = D_M \frac{d^2M}{dx^2} + 2I(x) - Q(x) \quad \textbf{(2)}$$

$$I(x) = E(x)K_{cat}\frac{S}{K_m^E + S} \quad \textbf{(3)}$$

$$Q(x) = \left(V_{max}^1 \frac{M}{K_m^1 + M} + V_{max}^2 \frac{M}{K_m^2 + M}\right) d_{cell}(x) \quad \textbf{(4)}$$

$$\frac{dE}{dt}(x) = \alpha \frac{M}{K_\alpha + M} d_{cell}(x) \quad \textbf{(5)}$$

$$\frac{d(d_{cell})}{dt}(x) = \mu_{max} \frac{M}{K_S + M} d_{cell}(x) \quad \textbf{(6)}$$

Details of the different variables and constants are summarized in Supplementary Table 1.

Parameter adjustment

We manually tuned these parameters so that the simulations fit both the dynamic and final densities of the corresponding experiments. We found the following values: μ_{max} = 0.27 h^{-1}, α_{coop} = 1.8E-24 mol.s^{-1}.$cell^{-1}$ (corresponds to the maximal DMD light intensity) and α_{cheat} = 1.5E-25 mol.s^{-1}.$cell^{-1}$ (corresponds to the promoter leaking and the minimal DMD light intensity).

Simulation

To solve the PDEs, we use a Python package called *scikit-fdiff* (https://scikit-fdiff.readthedocs.io). We chose the Crack-Nicholson scheme to compute the diffusion of molecules across a discretized space and used reflective boundaries. We use a simulation hook to compute non-linear terms and prevent negatives values. We ran all simulations on an Intel(R) Xeon(R) CPU E5-1650v4 3.60 GHz processor and 64 GB of RAM.

Supplementary Table 1. Summary of the model parameters for yeast metabolism and growth on sucrose.

Variable	Description	Unit	Initial value	Source	Reference
M	Hexose concentration	M	1.00E-08	Fixed experimentally	
S	Sucrose concentration	M	0.029	Fixed experimentally	
E	Invertase concentration	M	1.00E-35	Fixed experimentally	
α	Enzyme production rate	$mol.s^{-1}.cell^{-1}$	1.8E-24 or 1.5E-25	User defined	
d	Cell density	$cell.L^{-1}$	2.85E+09	Fixed experimentally	
I	Invertase activity	$M.s^{-1}$	-	Computed	
Q	Hexose consumption	$M.s^{-1}$	-	Computed	
D_S	Diffusion of sucrose	$m^2.s^{-1}$	6.10E-10	Fixed experimentally	[1]
D_M	Diffusion of hexoses	$m^2.s^{-1}$	7.60E-10	Fixed experimentally	[1]
K_{cat}	Kcat of monomeric Invertase	s^{-1}	4700	Fixed experimentally	[2]
μ_{max}	Maximal growth rate	s^{-1}	7.50E-05	User defined	
V_{max1}	Maximal consumption rate	$mol.s^{-1}.cell^{-1}$	4.18E-17	Fixed experimentally	[3]
V_{max2}	Maximal consumption rate	$mol.s^{-1}.cell^{-1}$	2.60E-17	Fixed experimentally	[3]
K_{m1}	Affinity constant for hexoses	M	0.0008	Fixed experimentally	[3]
K_{m2}	Affinity constant for hexoses	M	0.021	Fixed experimentally	[3]
K_s	Monod constant	M	0.00012	Fixed experimentally	[4]
K_m^E	Km of invertase	M	0.026	Fixed experimentally	[2]
K_α	Invertase production	M	1.00E-05	User defined	

Supplementary Table 2. List of yeast strains used in this study.

Name	HO locus	SUC2 locus	HIS locus	Nuclear marker	Background
yPH_428	N/A	N/A	N/A	N/A	BY4741
yPH_436	N/A	N/A	EL222-HIS3	N/A	BY4741
yPH_449	N/A	N/A	EL222-HIS3	HTB2::mApple-Kan	BY4741
yPH_457	N/A	ΔSUC2	EL222-HIS3	HTB2::mApple-Kan	BY4741
yPH_470	pC120-Venus-SUC2	ΔSUC2	EL222-HIS3	HTB2::mApple-Kan	BY4741
yPH_471	pC120-SUC2-P2A-Venus	ΔSUC2	EL222-HIS3	HTB2::mApple-Kan	BY4741
yPH_484	N/A	P2A-Venus	EL222-HIS3	HTB2::mApple-Kan	BY4741
yPH_536	pC120-SUC2-HairPinC09	ΔSUC2	EL222-HIS3	HTB2::mApple-Kan	BY4741
yPH_540	pC120GAL1-SUC2-HairPinC09	ΔSUC2	EL222-HIS3	HTB2::mApple-Kan	BY4741

Supplementary Table 3. **List of plasmids used in this study.** We built several “level 0” plasmids compatible with the Modular Cloning framework[5] so we could easily assemble transcriptional units in “level 1” plasmids. All plasmids and sequences are available upon request.

Name	Use	Plasmid type	MoClo part type	Description
pPH297	Repair fragment for homologous recombination (HR) with auxotrophic selection	x	x	EL222-HIS fragment by digestion
pPH330	PCR template for repair fragment for HR with antibiotic selection	FA6a	x	HTB2-mApple-Kan PCR
pPH162	Integration of optogenetically controlled genes	Expression cassette for CRIPSR/Cas9+gRNA	x	pML104-HO
pPH362	SUC2 deletion	Expression cassette for CRIPSR/Cas9+gRNA	x	pML104-SUC2
pPH374	SUC2 tagging with -P2A-Venus	Expression cassette for CRIPSR/Cas9+gRNA	x	pML104-SUC2
pPH296	PCR template for pC120 integration in a “level 0” (lvl0) entry vector	x	x	pC120 PCR
pPH349	PCR template for GAL1 minimal promoter to build p2RGal	x	x	pGAL1 PCR
pYTK97	Golden Gate assembly to construct “level 1” (lvl 1) MoClo plasmid	MoClo lvl0	2	pC120
pYTK111	Golden Gate assembly to construct lvl 1 MoClo plasmid	MoClo lvl0	3a	SUC2
pYTK112	Golden Gate assembly to construct lvl 1 MoClo plasmid	MoClo lvl0	3b	P2A
pYTK135	Golden Gate assembly to construct lvl 1 MoClo plasmid	MoClo lvl0	3	SUC2
pYTK137	Golden Gate assembly to construct lvl 1 MoClo plasmid	MoClo lvl0	2	pC120GAL1_2R
pYTK140	Golden Gate assembly to construct lvl 1 MoClo plasmid	MoClo lvl0	4a	HP: Hairpin mRNA degron C09
pYTK118	Repair fragment for HR with CRISPR	MoClo lvl1	x	pC120-SUC2-P2A-Venus
pYTK119	Repair fragment for HR with CRISPR	MoClo lvl1	x	pC120-SUC2-Venus
pYTK147	Repair fragment for HR with CRISPR	MoClo lvl1	x	pC120-SUC2-HP
pYTK151	Repair fragment for HR with CRISPR	MoClo lvl1	x	p2RGal-SUC2-HP

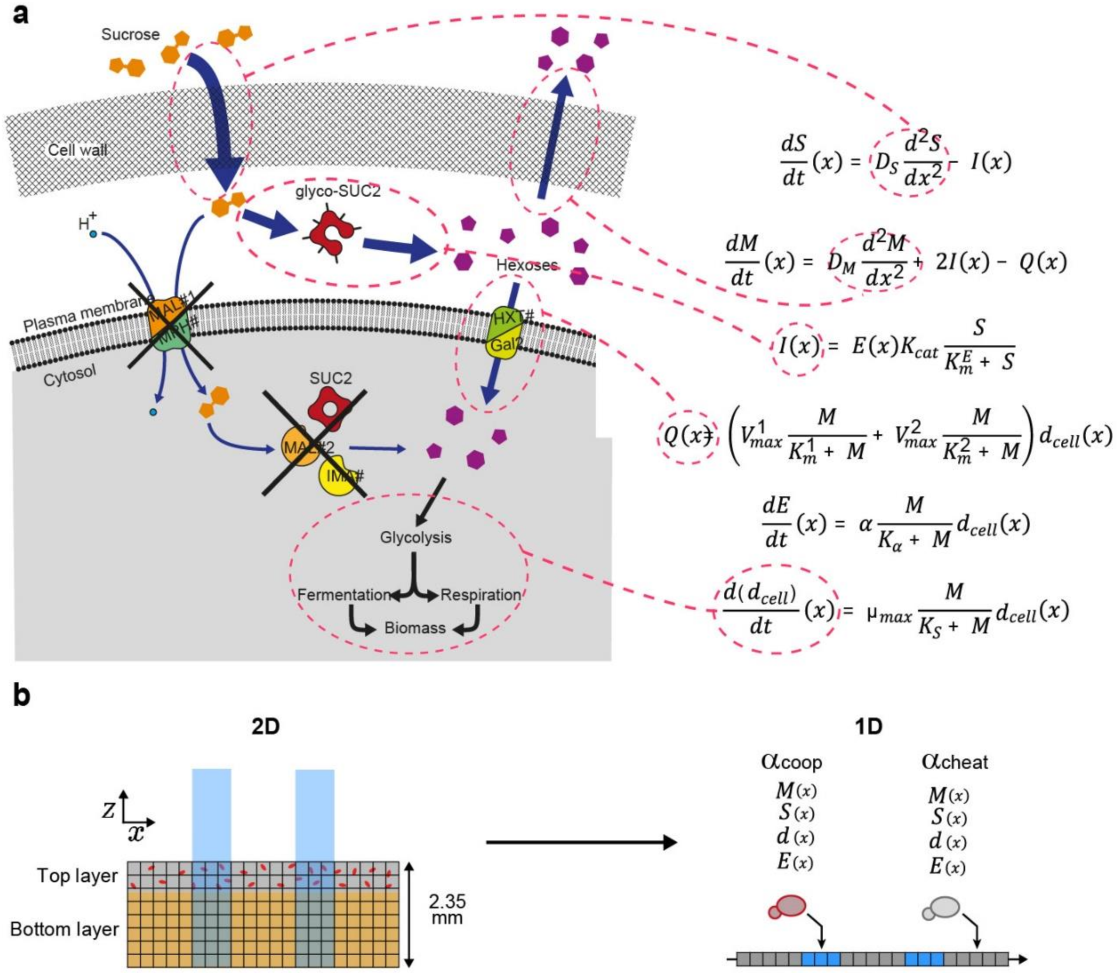

Supplementary Figure 1. Modelling yeast growth on sucrose. (**a**) *Saccharomyces cerevisiae* sucrose pathway and the corresponding set of partial differential equations used to model the system. (**b**) Schematic view of the model (left) and its dimensional reduction to a one-dimensional problem (right). The geometry of the gel allows us to assume that diffusion in the *z*-axis happens very rapidly compared to diffusion in the *x* and *y* directions: $\Delta Z = 2.35$ mm $<< \Delta X = \Delta Y = 53$ mm. We can thus simplify the model by considering the gel as homogeneous in the *z*-axis. We take dilution into account using the layer thicknesses (1.68 mm Phytagel and 0.67 mm agarose) to convert the sucrose concentration and cell density values.

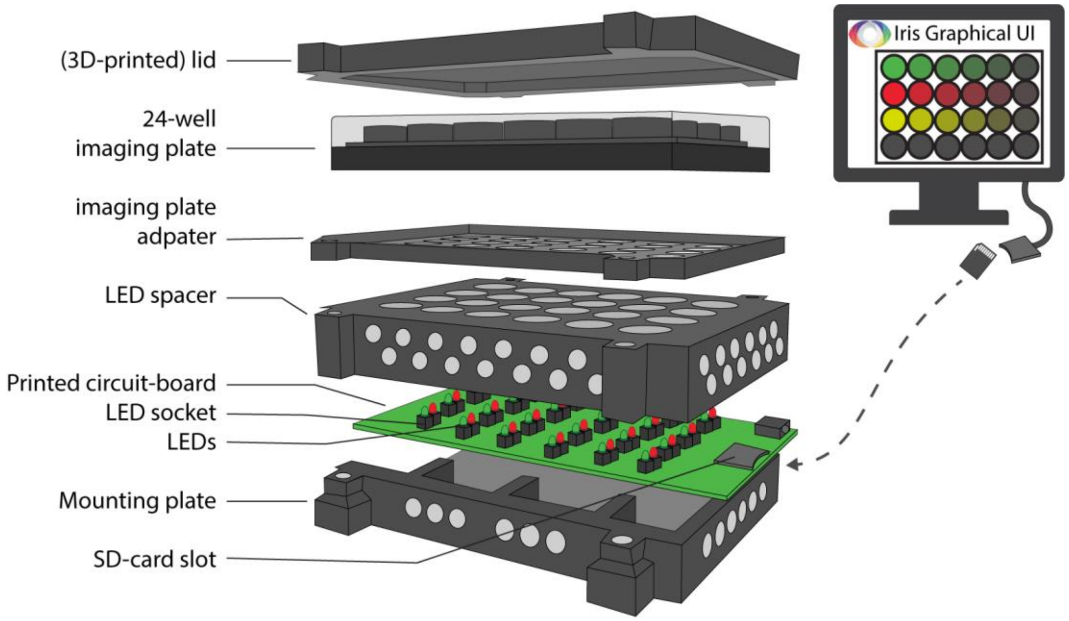

Supplementary Figure 2. 3D sketch of the Light Plate Apparatus[6] we used to activate SUC2 production with light (reproduced from[7]) . We routinely cultured yeast cells in 24-well plates and programmed the blue LEDs for each well to screen for optogenetic activation of SUC2. More details of the LPA can be found on the Tabor Lab's github (https://github.com/taborlab/LPA-hardware).

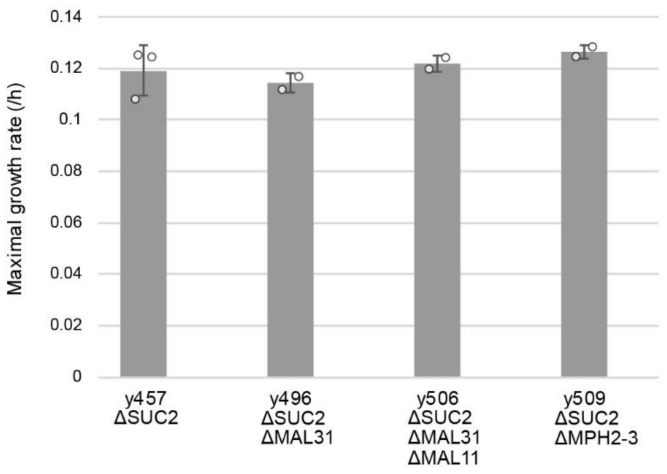

Supplementary Figure 3. Maximal growth rate of mutants of SUC2, MAL31, MAL11, and MPH2-3 in SC 1% sucrose obtained by measuring the increase in optical density over time.

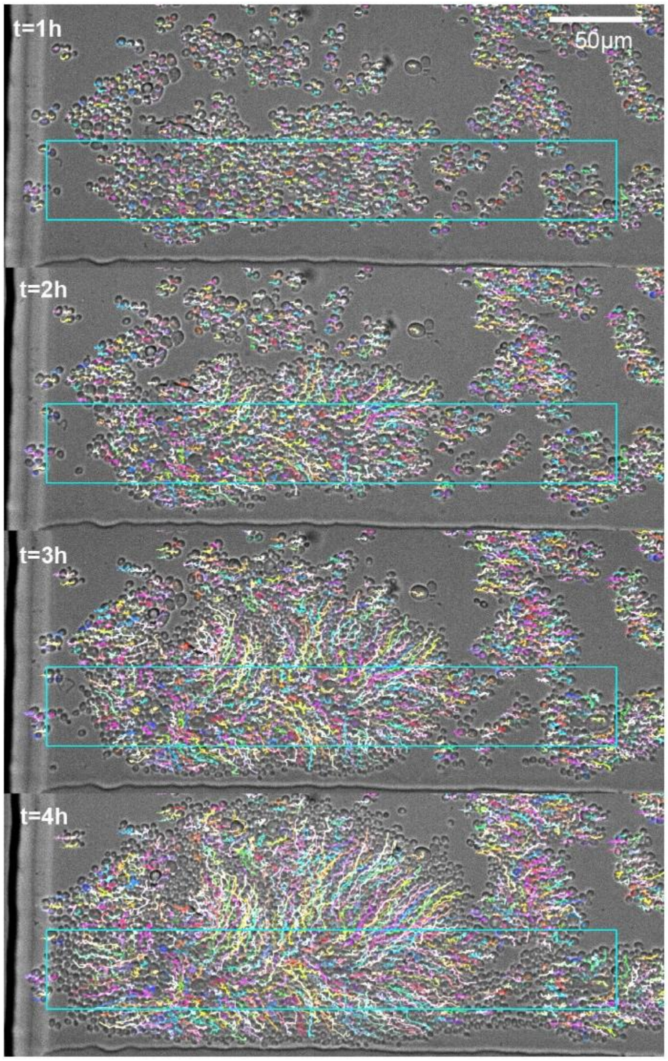

Supplementary Figure 4. Tracking of yeast cells (OptoSuc2) from Figure 3 (main text). Cells are growing in a microfluidic chamber perfused with 1% sucrose and a DMD was used to shine light on a given area (cyan rectangle). Tracking was performed with the TrackMate plugin[8] in ImageJ to confirm that growing cells push each other outside of the illumination area. Only high-quality trajectories starting at t = 0 h are plotted for better visibility. The (cyan) rectangle represents the area illuminated using the Mosaic (DMD) at 460 nm for 200 ms every 6 min.

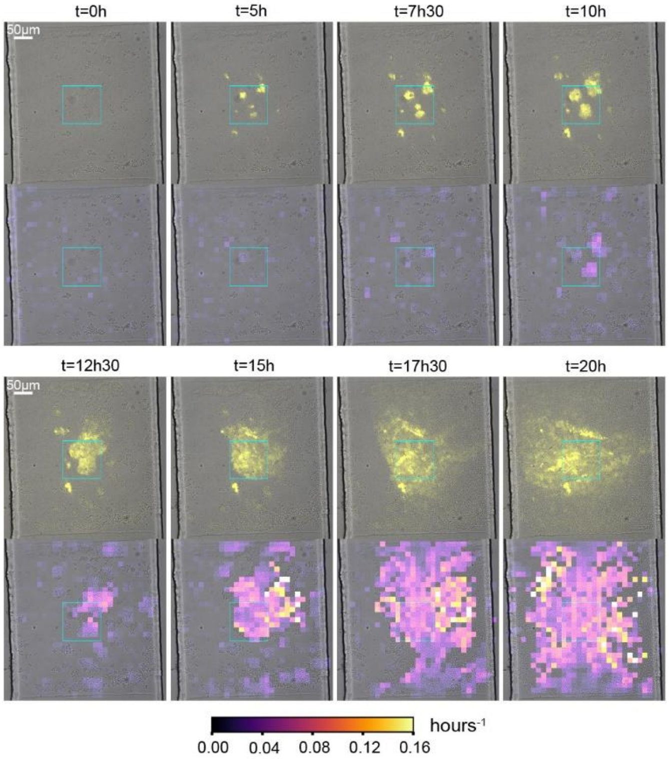

Supplementary Figure 5. Timelapse microscopy of SUC2-P2A-YFP strains (yPH_471) in a microfluidic chamber perfused with 1% sucrose. Cells within the cyan square were activated by light (typically ~50 cells). We show the time series of an overlay of brightfield and yellow fluorescence images (top) and the divergence map of local cell velocities (bottom) to identify regions where cells are growing. The cyan square represents the area illuminated using the Mosaic (DMD) at 460 nm (intensity 20%) for 250 ms every 6 min. This experiment shows that cell growth leads to a flow of cells outside of the illuminated area. These cells are still marked by fluorescence because of the long lifetime of YFP. Similarly, we expect that these cells still express functioning Suc2p in their periplasmic space, which would allow the cells to process sucrose and to grow. As a result, growth is rapidly not constrained to the illuminated area but invades the full area of the microfluidic chamber. The long lifetime of Suc2p (and YFP) prevented us from limiting the growth of cells to the illuminated area at such a small scale.

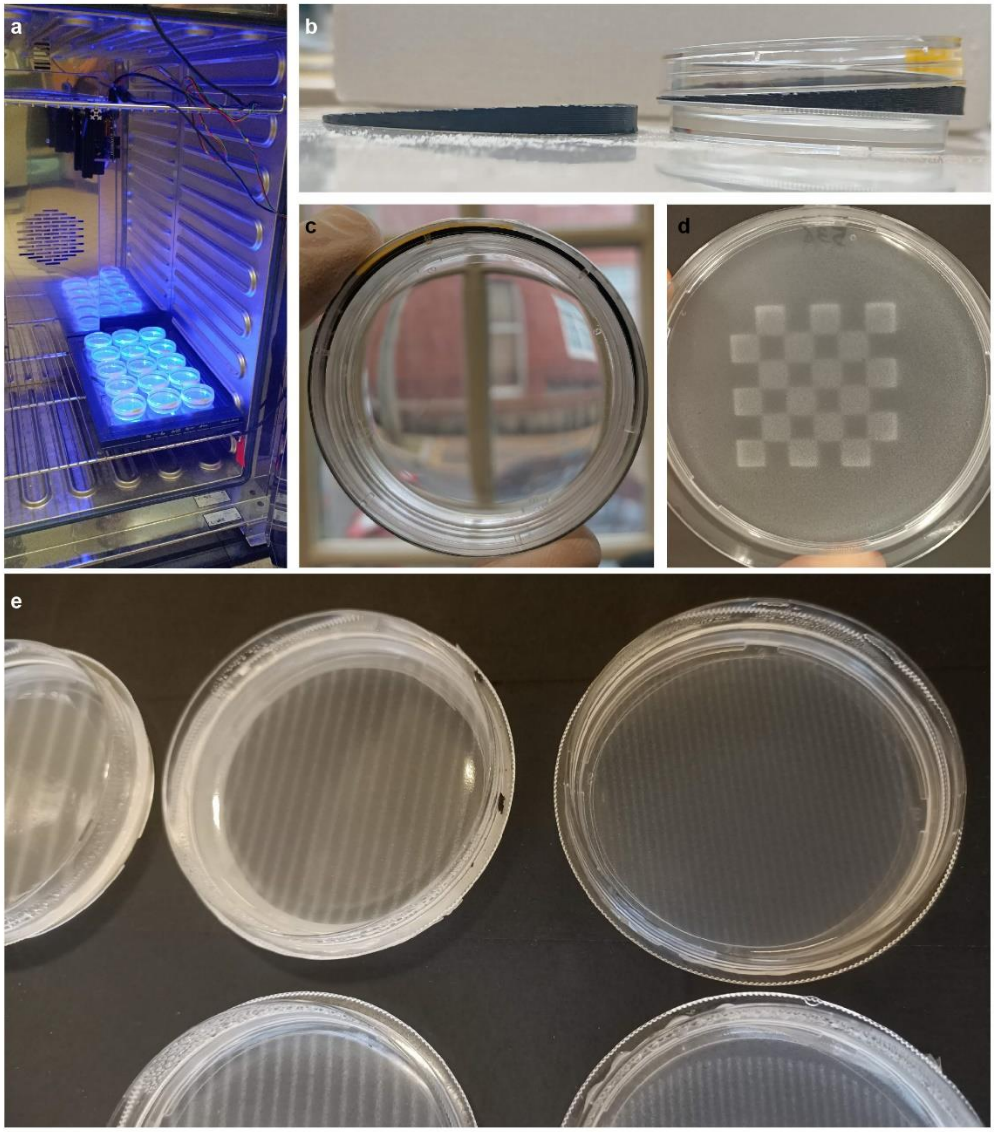

Supplementary Figure 6. Pictures of the OptoCube device and its application to activate growth at selected locations at the centimetre scale. (**a**) Interior of the OptoCube with the DMD (top) illuminating 15 plates placed on top of a scanner. The large incubator could accommodate a second DMD/scanner system, if required. (**b**) 3D printed part used to tilt the lids to 5° (see Supplementary Method 1). This avoids rapid drying of the plates and avoids direct reflection of the DMD on the plates. (**c**) Image of an agar plate containing a two-layer gel with a homogeneous yeast suspension before light induction. The agar plate is transparent and exhibits homogenous cell density. (**d-e**) Examples of the growth patterns obtained when plates were illuminated with arrays of lines (**d**) and a chess board pattern (**e**).

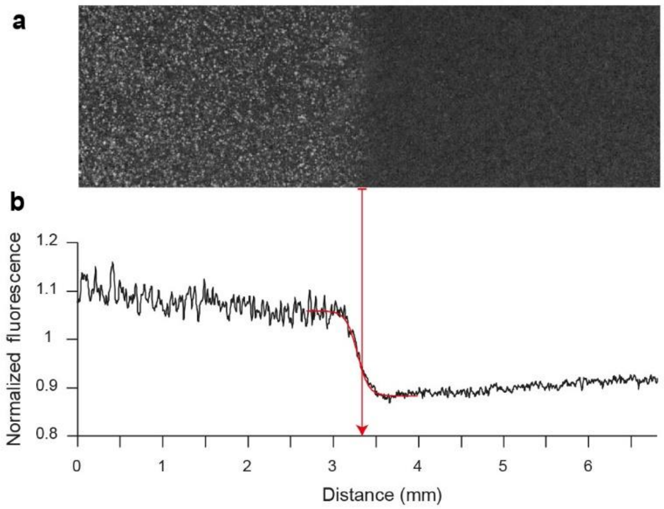

Supplementary Figure 7. Spatial resolution of the optogenetic induction of gene expression in the OptoCube. We applied a light pattern with a sharp transition from 100% to 0% of intensity for 24 h on a bilayered agar plate, with the top layer containing an optogenetic yeast strain that produces YFP under the control of the EL222/pC120 optogenetic system. (**a**) Normalized fluorescent image of the light pattern transition captured by a fluorescence macroscope showing the fluorescence of cell clumps and (**b**) quantification of the fluorescence intensity profile across the light-dark transition region. The normalization was performed by dividing the image by the image of a non-illuminated plate. The red line represents the fit of a sigmoid function $y=1/(1+\exp(-\lambda x))$ with parameter λ = 14.95 mm^{-1}. As shown in the figure, the transition is sharp and typically of the order of half a millimeter.

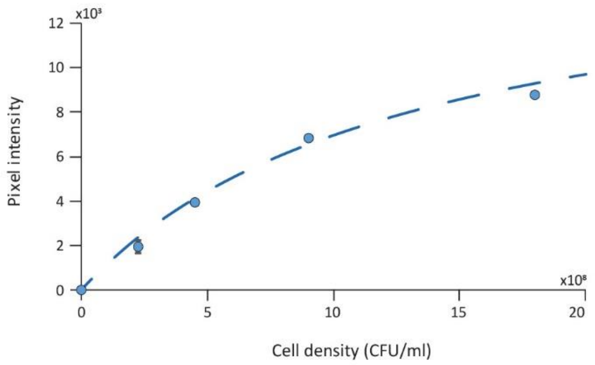

Supplementary Figure 8. **Calibration curve of the scanner** to determine the relationship between pixel intensity and cell density. We prepared agar plates with known cell densities in the top layer and measured the pixel intensities acquired by the scanner. We then fitted the data with the function $y = y_0 \frac{x}{x+x_0}$ with $y_0 = 1.6E4$ and $x_0 = 1.3E9$ and used this function to convert the pixel intensities into cell densities for our experiments. Round circles represent the mean and error bars represent the standard deviation of duplicates, and the fit is plotted as a dashed line.

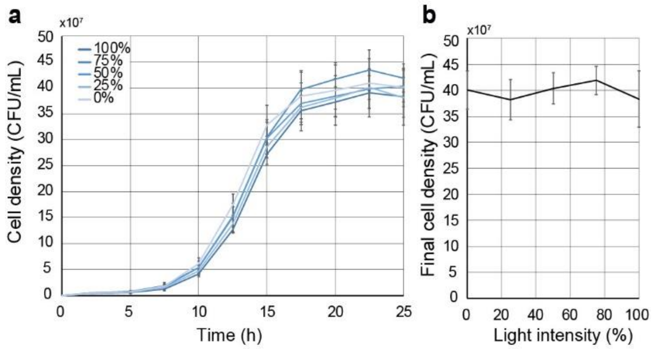

Supplementary Figure 9. **Growth of a homogeneous layer of cells**. We illuminated the entire surface of agar plates containing the WT strain and varied the light intensity from 0 to 100% (1.13 mW.cm$^{-2)}$. (**a**) Variation in the cell density as a function of time. Lines represent means of triplicate and error bars represent ± standard deviation. (**b**) Dependence of the final cell density on the light intensity at t = 25 h. No significant phototoxicity was observed in our system.

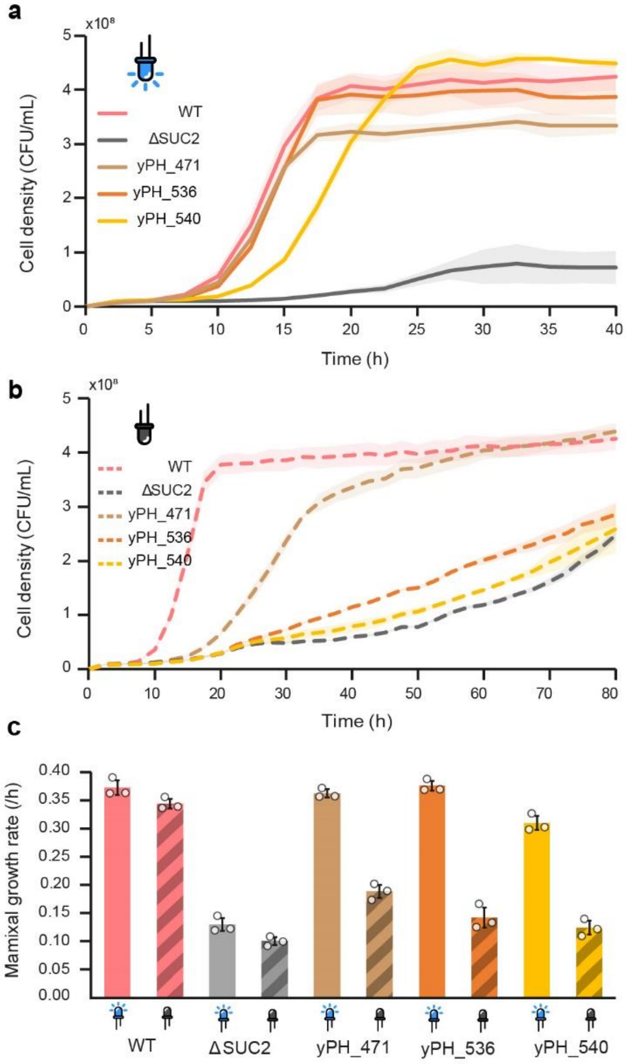

Supplementary Figure 10. Growth curves of different optogenetic strains with homogeneous illumination in the OptoCube obtained in 1% sucrose. (a, b) Growth at 100% light intensity (1.13mW/cm²) **(a)** and in the dark (control) (**b**). Lines represent the mean of triplicate experiments and the shaded areas represent ± one standard deviation. (**c**). From **a** and **b**, we extracted the maximal growth rate (error bars represent ± one standard deviation of triplicates) of the strains that were exposed or not to light. See also Figure 2, which shows the same growth rate differences were obtained in liquid culture.

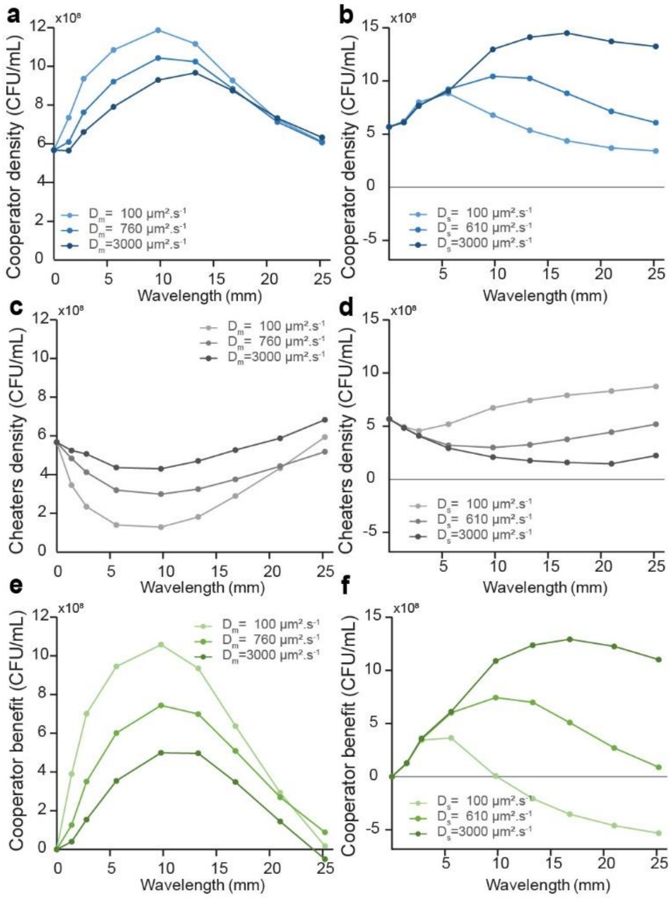

Supplementary Figure 11. **Numerical testing of the dependency of the diffusion coefficients of glucose and sucrose on the lower and larger cut-offs of the cooperator/cheater spatial filtering properties.** (**a, c, e**) The lower the diffusion of glucose, Dm, the smaller the lower cutoff λ_-, consistent with a shorter length-scale of diffusion of glucose, which limits cheaters' growth. The impact of the glucose diffusion coefficient on the larger cutoff was less pronounced. (**b, d, f**) The lower the diffusion coefficient of sucrose, Ds, the smaller the larger cut-off λ_+. This is consistent with sucrose diffusion setting the length-scale of competition within the cooperator domain. The sucrose diffusion coefficient had limited (if any) impact on the lower cutoff.

Supplementary references

1. Ribeiro, A. C. F. *et al.* Binary mutual diffusion coefficients of aqueous solutions of sucrose, lactose, glucose, and fructose in the temperature range from (298.15 to 328.15) K. *J. Chem. Eng. Data* **51**, 1836–1840 (2006).
2. Reddy, A. & Maley, F. Studies on identifying the catalytic role of glu-204 in the active site of yeast invertase. *J. Biol. Chem.* **271**, 13953–13958 (1996).
3. Reifenberger, E., Boles, E. & Ciriacy, M. Kinetic characterization of individual hexose transporters of *Saccharomyces cerevisiae* and their relation to the triggering mechanisms of glucose repression. *European Journal of Biochemistry* **245**, 324–333 (1997).
4. Snoep, J. L., Mrwebi, M., Schuurmans, J. M., Rohwer, J. M. & Teixeira de Mattos, M. J. Control of specific growth rate in *Saccharomyces cerevisiae*. *Microbiology* **155**, 1699–1707 (2009).
5. Lee, M. E., DeLoache, W. C., Cervantes, B. & Dueber, J. E. A Highly characterized yeast toolkit for modular, multipart assembly. *ACS Synth. Biol.* **4**, 975–986 (2015).
6. Gerhardt, K. P. *et al.* An open-hardware platform for optogenetics and photobiology. *Scientific Reports* **6**, (2016).
7. Pouzet, S. *et al.* The Promise of optogenetics for bioproduction: Dynamic control strategies and scale-up instruments. *Bioengineering* **7**, 151 (2020).
8. Tinevez, J.-Y. *et al.* TrackMate: An open and extensible platform for single-particle tracking. *Methods* **115**, 80–90 (2017).
