# Supplementary material for: Optogenetic spatial patterning of cooperation in yeast populations
Source: Nat Commun. 2024 Jan 2;15:75. doi: 10.1038/s41467-023-44379-5 (PMC10761962; doi:10.1038/s41467-023-44379-5)
Supplement: Supplementary file 3 — Description of Additional Supplementary Files [file 41467_2023_44379_MOESM3_ESM.pdf]

Description of Additional Supplementary Files

File Name: Supplementary Movie 1
Description: Spatial control of yeast growth in a microfluidic chamber. OptoSUC2 yeast cells grow in chambers of 400 μm^2 and are perfused via two large channels on the sides of the chambers. The height of the chamber (3.5 µm) ensures that cells grow as a monolayer. A digital micromirror device is used to project a pattern of light in the field of view (blue rectangle illuminated at 460 nm for 200 ms every 6 min). Time-lapse bright-field images were analyzed through PIV (particle image velocimetry) to generate a displacement vector map (left). We also computed the divergence map of the vector field, which is a proxy for local cell growth (right).

File Name: Supplementary Movie 2
Description: Reproducing a picture with optogenetics-enabled yeast growth. We projected an image of Maud Menten as a tribute to her work on the Michaelis-Menten enzymatic kinetic equation using invertase as a model. Her portrait was projected from the DMD onto a Petri dish containing OptoSuc2 cells for 45 h and the plate was scanned periodically. The yeast grew in a pattern mirroring the portrait. The first image of the timelapse was subtracted as the background to obtain the movie on the left. On the right, inverted images of the resulting yeast growth reveal the developed image of Maud Menten through OptoSUC2 lightinduced growth.

File Name: Supplementary Movie 3
Description: Varying the size of the cooperator domain impacts the density of cooperators. We projected single lines of light with varying widths and observed the growth of cells in the cooperating (illuminated) and cheating (dark) domains over time. Background subtraction was performed using the first image in the timelapse. Increasing the width of the cooperator domain both decreased the final density of cooperators at the center of the line and increased the density of cheaters at the frontier of the dark and illuminated domains. Initially, hexoses are produced everywhere in the cooperating domain, which promotes the growth of cooperating cells, decreases the sucrose concentration, and creates a source of hexose (public good). This leads to competition for glucose and an increase in the density of cheaters located at the frontiers of the cooperating domain. Within large cooperating domains, competition for sucrose leads to a decay in the sucrose concentration towards the center and an increase in the density of cooperating cells at the frontiers with the cheater (dark) domain.

File Name: Supplementary Movie 4
Description: Periodic patterning of cooperator and cheater domains gives a quantitative measurement of cooperation and competition length-scales. We projected periodic lines of light of various widths, maintaining a constant ratio between dark and illuminated domains (75% dark, 25% illuminated). The first image was used as background measurement. Cooperators and cheaters grow in their respective domains, but the cooperator cells have a marked fitness advantage. Below the lower cutoff (λ- ~ 7 mm), the cooperator domains are too small to retain all of the glucose they produce for their own profit and the cheater cells in the dark areas can grow. Above the larger cutoff (λ+ ~ 17 mm), the cooperator domain is too large to be sustainable everywhere given the limited influx of sucrose from the frontier. As a result, the cells in the central part of the cooperator domain are in competition for both sucrose and glucose (self-competition), which leads to a lower final cooperator cell density.
