# Supplementary material for: Optogenetic spatial patterning of cooperation in yeast populations
Source: Nat Commun. 2024 Jan 2;15:75. doi: 10.1038/s41467-023-44379-5 (PMC10761962; doi:10.1038/s41467-023-44379-5)
Supplement: Supplementary file 8 — Reporting Summary [file 41467_2023_44379_MOESM8_ESM.pdf]

nature portfolio

Corresponding author(s): NCOMMS-23-21676

Last updated by author(s): Dec 5, 2023

Reporting Summary

Nature Portfolio wishes to improve the reproducibility of the work that we publish. This form provides structure for consistency and transparency in reporting. For further information on Nature Portfolio policies, see our Editorial Policies and the Editorial Policy Checklist.

Statistics

For all statistical analyses, confirm that the following items are present in the figure legend, table legend, main text, or Methods section.

n/a	Confirmed	
☐	☒	The exact sample size (*n*) for each experimental group/condition, given as a discrete number and unit of measurement
☒	☐	A statement on whether measurements were taken from distinct samples or whether the same sample was measured repeatedly
☒	☐	The statistical test(s) used AND whether they are one- or two-sided<br>*Only common tests should be described solely by name; describe more complex techniques in the Methods section.*
☒	☐	A description of all covariates tested
☒	☐	A description of any assumptions or corrections, such as tests of normality and adjustment for multiple comparisons
☐	☒	A full description of the statistical parameters including central tendency (e.g. means) or other basic estimates (e.g. regression coefficient) AND variation (e.g. standard deviation) or associated estimates of uncertainty (e.g. confidence intervals)
☒	☐	For null hypothesis testing, the test statistic (e.g. *F*, *t*, *r*) with confidence intervals, effect sizes, degrees of freedom and *P* value noted<br>*Give P values as exact values whenever suitable.*
☒	☐	For Bayesian analysis, information on the choice of priors and Markov chain Monte Carlo settings
☒	☐	For hierarchical and complex designs, identification of the appropriate level for tests and full reporting of outcomes
☒	☐	Estimates of effect sizes (e.g. Cohen's *d*, Pearson's *r*), indicating how they were calculated

Our web collection on statistics for biologists contains articles on many of the points above.

Software and code

Policy information about availability of computer code

Data collection	Data were collected through open source software to control image acquisition through a scanner or to perform time lapse microscopy. We used CyberSco.Py v1 (open source smart microscopy software from our team. See article here 10.1038/s41598-022-15207-5 and the code here: https://github.com/Lab513/CyberSco.Py) and a home made python based software (see Supplementary Information and Github repository (https://github.com/Lab513/DIY_OptoCube)
Data analysis	Most of the data analysis did not require advanced software. We used the open source JPIV software (https://github.com/eguvep/jpiv/). Code used to perform numerical simulations was done with Python and Jupyter notebook and are available on GitHub (https://github.com/Lab513/Code_YeastCoop).

For manuscripts utilizing custom algorithms or software that are central to the research but not yet described in published literature, software must be made available to editors and reviewers. We strongly encourage code deposition in a community repository (e.g. GitHub). See the Nature Portfolio guidelines for submitting code & software for further information.

Data

Policy information about availability of data

All manuscripts must include a data availability statement. This statement should provide the following information, where applicable:

- Accession codes, unique identifiers, or web links for publicly available datasets
- A description of any restrictions on data availability
- For clinical datasets or third party data, please ensure that the statement adheres to our policy

All Data used to generate figures (main text and supplementary information) are availabe on a Zenodo repository http://dx.doi.org/10.5281/zenodo.10260274. All materials (plasmids, strains) are available upon reasonable request.

Research involving human participants, their data, or biological material

Policy information about studies with human participants or human data. See also policy information about sex, gender (identity/presentation), and sexual orientation and race, ethnicity and racism.

Reporting on sex and gender	NA
Reporting on race, ethnicity, or other socially relevant groupings	NA
Population characteristics	NA
Recruitment	NA
Ethics oversight	NA

Note that full information on the approval of the study protocol must also be provided in the manuscript.

Field-specific reporting

Please select the one below that is the best fit for your research. If you are not sure, read the appropriate sections before making your selection.

☒ Life sciences ☐ Behavioural & social sciences ☐ Ecological, evolutionary & environmental sciences

For a reference copy of the document with all sections, see nature.com/documents/nr-reporting-summary-flat.pdf

Life sciences study design

All studies must disclose on these points even when the disclosure is negative.

Sample size	Experiments were done on large number of cells and were very reproducible from one experiment to another. This indicated that the samples size were large enough to give a precise estimate of the average behaviour of the cell populations.
Data exclusions	No data were excluded from analysis.
Replication	All experiments have been replicated several times (see figure legends for details).
Randomization	We performed experiments on microbial communities in well controlled conditions in which spatial groups are created by external light inductions. Randomization is not needed and cannot be used efficiently in such a settings where cells are not randomly assigned to experimental groups.
Blinding	Blinding was not considered in our study since the influence of the operator on how yeast cells behave/ respond was limited (automated, unsupervised image acquisition) on the image analysis and controls were run throughout the experiments to check for analysis output.

Reporting for specific materials, systems and methods

We require information from authors about some types of materials, experimental systems and methods used in many studies. Here, indicate whether each material, system or method listed is relevant to your study. If you are not sure if a list item applies to your research, read the appropriate section before selecting a response.

Materials & experimental systems

n/a	Involved in the study
☒	☐ Antibodies
☒	☐ Eukaryotic cell lines
☒	☐ Palaeontology and archaeology
☒	☐ Animals and other organisms
☒	☐ Clinical data
☒	☐ Dual use research of concern
☒	☐ Plants

Methods

n/a	Involved in the study
☒	☐ ChIP-seq
☒	☐ Flow cytometry
☒	☐ MRI-based neuroimaging
